# Effect of vaginal probiotics containing *Lactobacillus casei rhamnosus* (Lcr regenerans) on vaginal dysbiotic microbiota and pregnancy outcome, prospective, randomized study

**DOI:** 10.1038/s41598-023-34275-9

**Published:** 2023-05-02

**Authors:** Ljubomir Petricevic, Ingo Rosicky, Herbet Kiss, Nina Janjic, Ulrike Kaufmann, Iris Holzer, Alex Farr

**Affiliations:** Division of Obstetrics and Feto-Maternal Medicine, Department of Obstetrics and Gynecology, Medical University of Vienna, Waehringer Guertel 18-20, 1090 Vienna, Austria

**Keywords:** Urogenital reproductive disorders, Clinical microbiology

## Abstract

The intermediate bacterial microbiota is a heterogeneous group that varies in the severity of the dysbiosis, from minor deficiency to total absence of vaginal *Lactobacillus spp*. We treated women with this vaginal dysbiosis in the first trimester of pregnancy using a vaginally applied lactobacilli preparation to restore the normal microbiota in order to delay the preterm delivery rate. Pregnant women with intermediate microbiota of the vagina and a Nugent score of 4 were enrolled in two groups: intermediate vaginal microbiota and a Nugent score of 4 *with* lactobacilli (IMLN4) and intermediate vaginal microbiota and a Nugent score of 4 *without* lactobacilli (IM0N4), with and without vaginal lactobacilli at baseline, respectively. Half of the women in each group received the treatment. Among women without lactobacilli (the IM0N4 group), the Nugent sore decreased by 4 points only in the women who received treatment, and gestational age at delivery and neonatal birthweight were both significantly higher in the treated subgroup than in the untreated subgroup (*p* = 0.047 and *p* = 0.016, respectively). This small study found a trend toward a benefit of treatment with vaginal lactobacilli during pregnancy.

## Introduction

Despite progress in perinatology, prevention of preterm delivery (PTD) is still a leading problem in obstetrics^1^. Healthy vaginal microbiota, dominated by *Lactobacillus species* (spp.), is a significant protective factor against vaginal infections that are potentially associated with PTD^2^. Depletion of vaginal *Lactobacillus* spp. enhances anaerobic bacterial growth, which can subsequently progress to vaginal dysbiosis and bacterial vaginosis (BV)^3^. Since treatment of abnormal vaginal microbiota has been reported to have a positive effect in pregnant women, BV is widely accepted as a risk factor for PTD^4^. Although antibiotic therapy reduces the risk of PTD in pregnant women with abnormal vaginal microbiota, the use of prophylactic antibiotics in the general obstetric population is still under discussion^5,6^.

Currently, there is insufficient evidence on the natural history of intermediate vaginal microbiota to determine its prognosis^7^. This dysbiosis constitutes a widely heterogeneous group that shows a high degree of variability in the presence of *Lactobacillus* spp. and the vagina may or may not contain anaerobic bacteria^8,9^. A meta-analysis conducted in 2007 found that the intermediate vaginal microbiota was not associated with PTD, perinatal mortality, or maternal or neonatal infection^10^. In contrast, Hay et al.^11^ reported a high rate of late miscarriage (16–24 gestational weeks) in pregnant women harboring vaginal intermediate microbiota detected before 16 weeks of gestation. Additionally, other authors have reported a correlation between PTD and partial BV, which is visually defined as a smear with streaks of BV within wide areas of normal microbiota^12^.

We have observed a correlation between intermediate microbiota detected in first trimester of pregnancy and PTD. As we previously reported, intermediate microbiota was identified based on the observation of Gram-stained smears in 6.3% of the women screened^13^. In more than half of the smears obtained, a Nugent score of 4 was observed, and in two-thirds of these smears, lactobacilli were present. The absence of colonizing bacteria, including *Lactobacillus* spp., was reported in one-third of women with a Nugent score of 4. Notably, we found that the PTD rate was significantly lower in women with vaginal lactobacilli (OR: 0.34, 95% CI 0.21–0.55; *p* < 0.001). Using *Lactobacillus casei rhamnosus* (Lcr regenerans) after the use of antibiotics for the treatment of BV helps to restore the vaginal microbiota^14^. While depletion of lactobacilli and enhanced anaerobic bacterial growth are distinct features of BV, treatment with antibiotics also reduces the amount of *Lactobacillus* spp.^14^. This dysbiosis resembles the intermediate state, with a Nugent score of 4, if depletion of lactobacilli occurs.

We conducted a trial to evaluate the effects of treating women with vaginal dysbiosis, an intermediate vaginal microbiota during the first trimester of pregnancy, using vaginally applied lactobacilli to restore the normal microbiota and reduce the rate of PTD.

## Results

**Figure 1:**
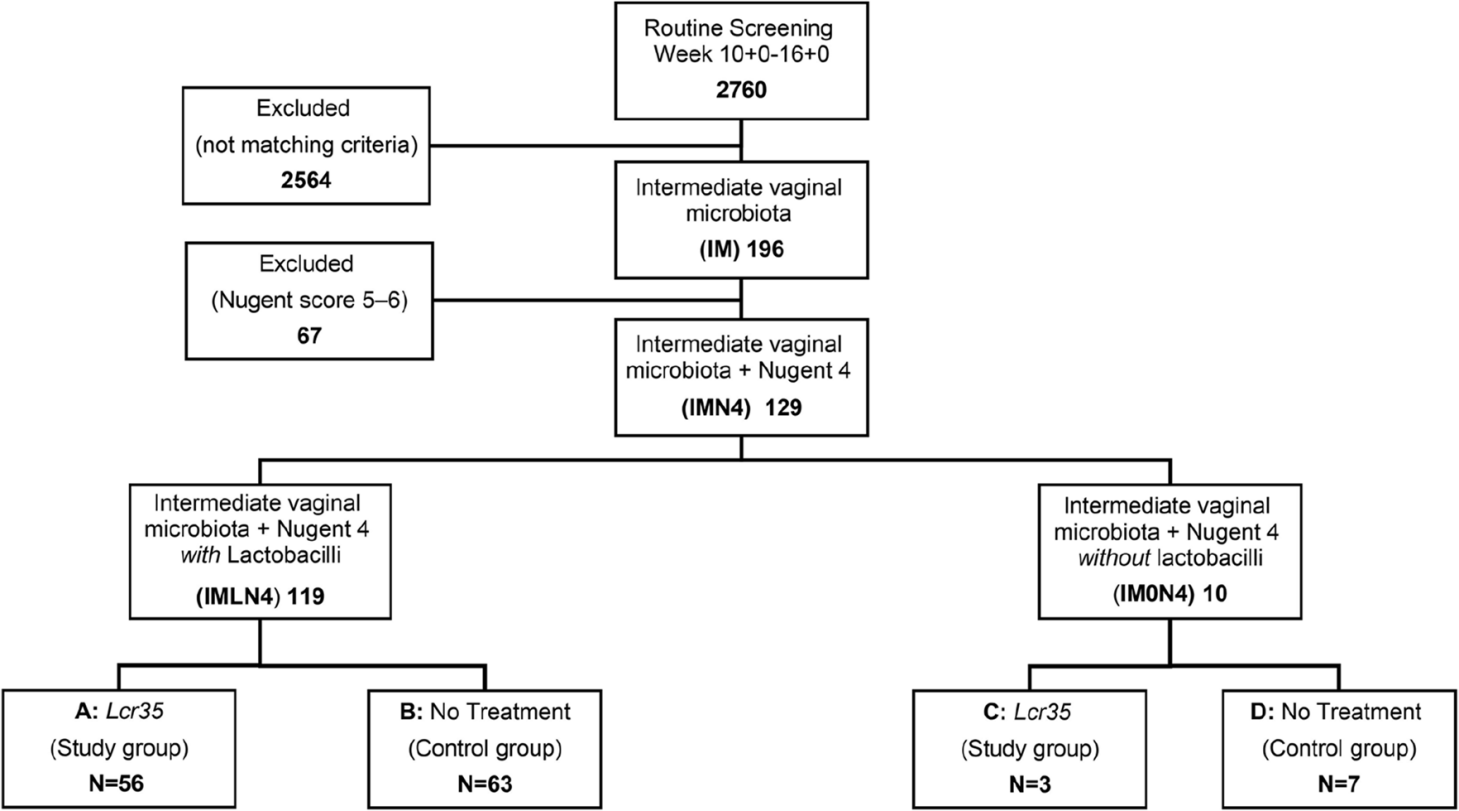
CONSORT diagram and flow chart showing the allocation of 129 pregnant women with Nugent scores of 4 on the baseline vaginal smear to treatment and control groups.

We enrolled 129 women with an intermediate vaginal microbiota and a Nugent score of 4, of whom 119 (92.2%) with vaginal lactobacilli were assigned to the intermediate vaginal microbiota and a Nugent score of 4 *with* lactobacilli (IMLN4) group, and 10 (7.8%) without vaginal lactobacilli were assigned to the intermediate vaginal microbiota and a Nugent score of 4 *without* lactobacilli (IM0N4) group. The CONSORT diagram is shown in Fig. 1. In ten patients from the IMLN4 group neither control swab nor delivery data could be obtained. The loss to follow-up has not occurred in IM0N4 group, resulting in a total of 119 pregnant women being included in this study. We found no statistically significant differences in maternal characteristics and perinatal outcomes between the 109 pregnant women of IMLN4 and 10 of IM0N4 groups, as shown in Table 1. Maternal age at delivery, parity, delivery mode, as well as umbilical cord pH and APGAR after delivery, were similar in both groups.

**Table 1:** Maternal characteristics and perinatal outcomes of the 119 women in the study, according to the presence (IMLN4) or absence (IM0N4) of lactobacilli in the vaginal smear at baseline.

	IMLN4 (N = 109)	IM0N4 (N = 10)	p value
Age at delivery (years)	32.5 ± 5.7	32.3 ± 6.7	0.885^a^
Obstetric history			
Previous pregnancies	3 (2–4)	2.5 (1–3)	0.591^b^
Previous deliveries	85 (71.4%)	5 (50%)	0.168^c^
Previous stillbirths	41 (34.5%)	2 (20%)	0.494 ^c^
Previous miscarriages	9 (7.6%)	2 (20%)	0.204 ^c^
Delivery mode			
Vaginal	49 (45.0%)	5 (50%)	> 0.999^c^
Caesarean section	51 (46.8%)	5 (50%)	
Instrumental	9 (8.3%)	0 (0%)	
Live birth	108 (99.1%)	10 (100%)	> 0.999^c^
Apgar score			
At 1 min	9 (8–9)	9 (9–9)	0.697^b^
At 5 min	10 (10–10)	10 (9–10)	0.540^b^
At 10 min	10 (10–10)	10 (10–10)	0.425^b^
Umbilical cord pH	7.26 ± 0.09	7.32 ± 0.08	0.081^a^

Results are presented as count (%), mean ± standard deviation, or median (interquartile range).

^a^Independent samples t-test.

^b^Mann-Whitney U test.

^c^Fisher’s exact test.

**Figure 2:**
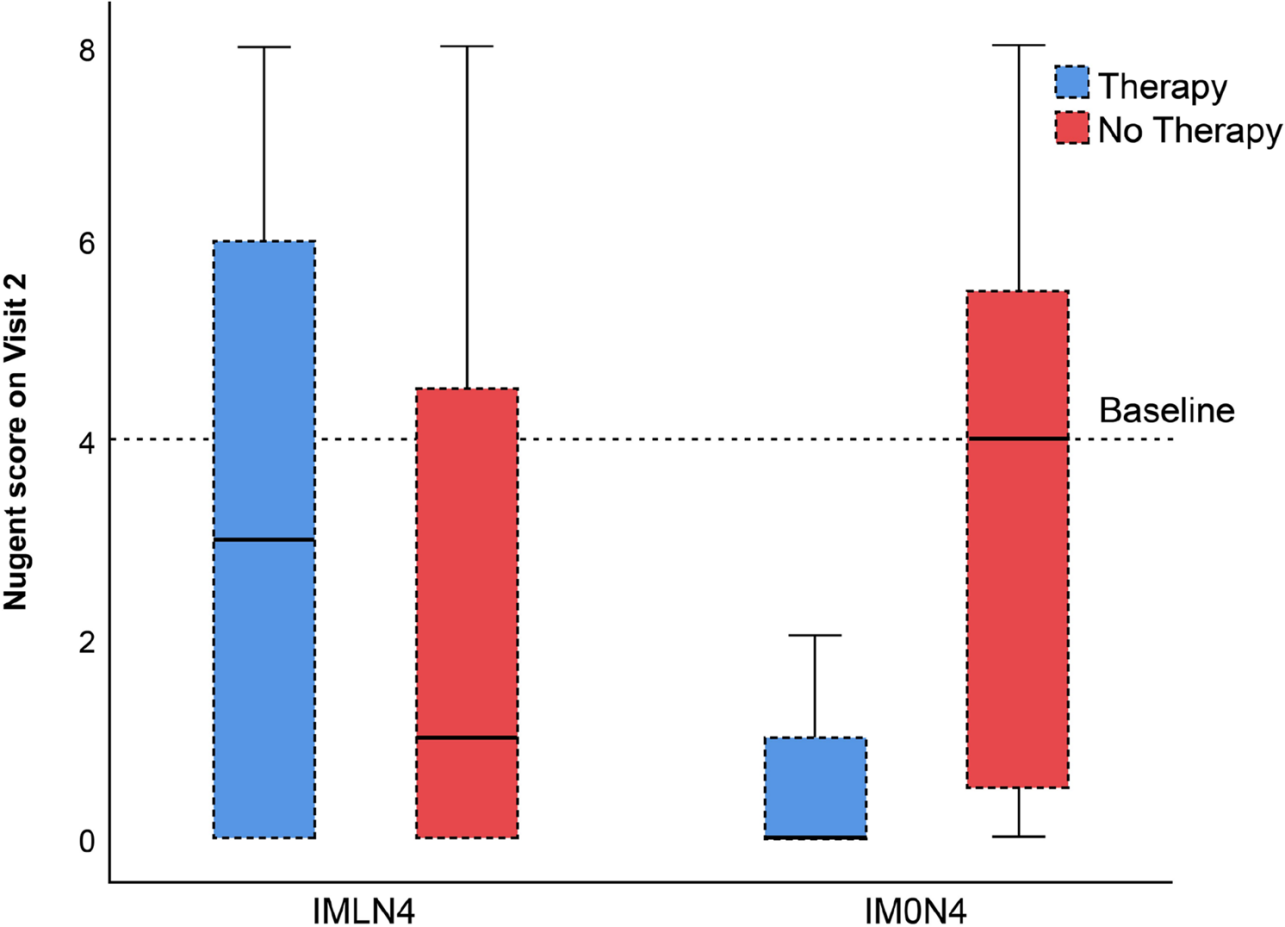
Box plots of the follow-up Nugent scores of 119 pregnant women with an intermediate vaginal microbiota and a Nugent score of 4 with lactobacilli (IMLN4) and pregnant women with an intermediate vaginal microbiota and a Nugent score of 4 without lactobacilli (IM0N4) on the baseline vaginal smear, *with* (subgroups A and C, respectively) and *without* (subgroups B and D, respectively) probiotic treatment during pregnancy.

In the comparison of the subgroups with and without treatment, we observed a low decrease in the Nugent score among both subgroups (A and B) among women with lactobacilli (IMLN4). Normal vaginal microbiota was achieved in 26 of 51 women (51%) in subgroup A (treatment group) and 34 of 58 women (58%) in subgroup B (control group), which was a nonsignificant difference (p = 0.438). Among women without lactobacilli (IM0N4), median Nugent score decreased by 4 points in the treatment group (subgroup C) but did not change in the control group (subgroup D). However, due to the small sample size of the IM0N4 group, this difference was not statistically significant (*p* = 0.233). The restoration to a normal microbiota was achieved in all 3 women (100%) in subgroup C, compared to 3 of 7 women (43%) in subgroup D (*p* = 0.2). Score differences are shown in Table 2. Total change of Nugent score from baseline regarding receipt of therapy or not, and compared between all subgroups is presented in Fig. 2.

**Table 2:** Vaginal microbiota among the 119 pregnant women in the study according to presence (IMLN4) or absence (IM0N4) of lactobacilli on vaginal smear at baseline, and whether they received (subgroups A and C) or did not receive (subgroups B and D) probiotic treatment during pregnancy.

	IMLN4			IM0N4		
	Subgroup A (N = 51)	Subgroup B (N = 58)	*p*	Subgroup C (N = 3)	Subgroup D (N = 7)	*p*
Nugent score at baseline	4	4		4	4	
Nugent score at follow-up	3 (0–6)	1 (0–4.5)	0.197^b^	0 (0–2)	4 (0–7)	0.233^b^
Nugent score change	− 1 (− 4 to 2)	− 3 (− 4 to 0.5)	0.197^b^	− 4 (− 4 to − 2)	0 (0–2)	0.233^b^
Preterm delivery	4 (7.8%)	3 (5%)	0.703^d^	0 (0%)	2 (29%)	> 0.999^d^
Gestational age at delivery (weeks)	38.5 ± 2.5	39.1 ± 1.5	0.163^a^	40.1 ± 0.4	37.1 ± 2.8	0.047^a^
Birthweight (g)	3214 ± 682	3305 ± 558	0.446^a^	3941 ± 329	2838 ± 816	0.016^a^

Results are presented as count (%), mean ± standard deviation, or median (interquartile range).

^a^Independent samples t-test.

^b^Mann-Whitney U test.

^c^Pearson chi square test.

^d^Fisher’s exact test.

The PTD rates, mean gestational age at birth, and birthweight among women with lactobacilli (IMLN4) were comparable and did not show any difference between subgroups A and B. In the IM0N4 group, the rate of PTD was higher in subgroup C than in subgroup D; however, the difference between groups was not statistically significant (*p* > 0.99). The mean gestational age at delivery and neonatal birth weight were significantly higher in women who received treatment with Lcr regenerans (subgroup C) (40.1 ± 0.4 weeks; 3941 ± 329 g) than in those who did not (subgroup D) (37.1 ± 2.8 weeks; 2838 ± 816 g) (*p* = 0.047 and *p* = 0.016, respectively).

**Figure 3:**
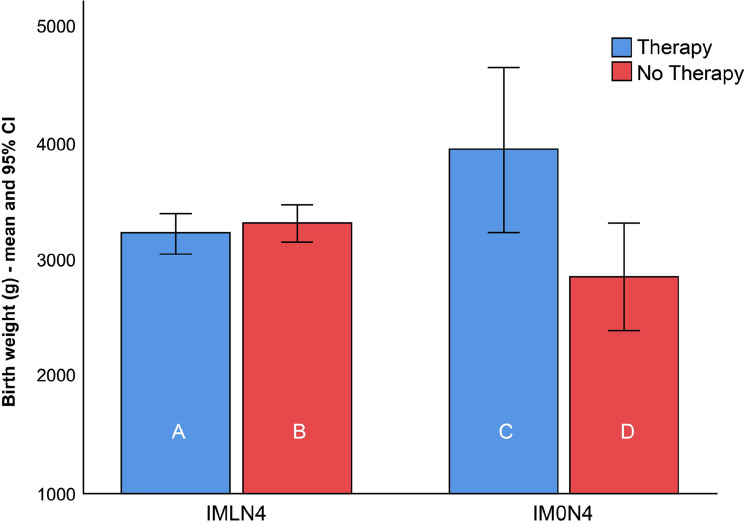
Box plots of the birthweight of 119 neonates born to women with an intermediate vaginal microbiota and a Nugent score of 4 with lactobacilli (IMLN4) and pregnant women with an intermediate vaginal microbiota and a Nugent score of 4 without lactobacilli (IM0N4) on the baseline vaginal smear, *with* (subgroups A and C) and *without* (subgroups B and D) probiotic treatment during pregnancy.

**Figure 4:**
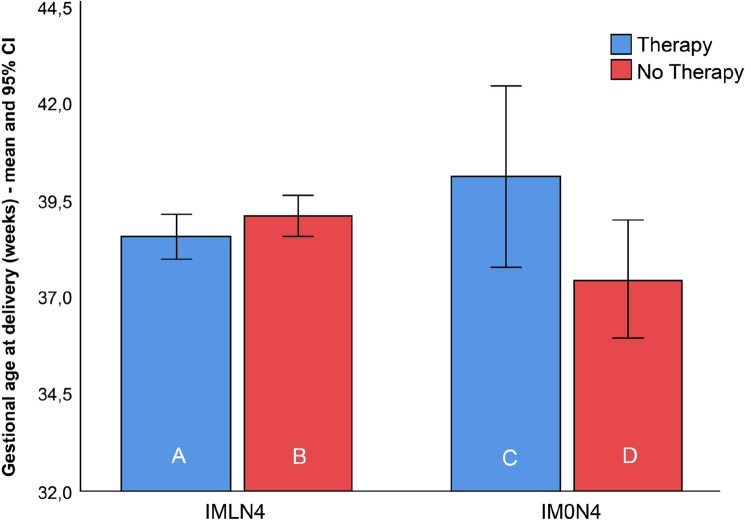
Box plots of the gestational age at delivery of 119 neonates born to women with an intermediate vaginal microbiota and a Nugent score of 4 with lactobacilli (IMLN4) and pregnant women with an intermediate vaginal microbiota and a Nugent score of 4 without lactobacilli (IM0N4) on the baseline vaginal smear, *with* (subgroups A and C) and *without* (subgroups B and D) probiotic treatment during pregnancy.

The summarized results are shown in Table 2. Box plots of the gestational age and birthweight at delivery of 119 neonates born to women with (IMLN4) and without (IM0N4) lactobacilli on the baseline vaginal smear, receiving therapy or not, are presented in Figs. 3 and 4.

## Discussion

The potential benefit of vaginally applying *Lactobacillus casei rhamnosus* (Lcr regenerans) after antibiotic treatment for BV has already been reported^14,15^. Moreover, the absence of vaginal lactobacilli and any bacterial colonization has been reported to increase the risk of PTD in women with intermediate microbiota^13^. This study evaluated the effect of Lcr regenerans on the intermediate vaginal microbiota in pregnant women with a Nugent score of 4, with particular interest in the effect of treatment in women with a total absence of *Lactobacillus* species in first trimester of pregnancy*.*

Although current literature suggests that women with PTD have a reduced presence of lactobacilli as part of the vaginal microbiome, increased bacterial diversity, and low vaginal levels of beta-defensin-2^16^, a recent systematic review and meta-analysis on the use of probiotic microorganisms during pregnancy reported neither positive nor negative effects on perinatal outcomes^17^. However, the majority of the studies included in the meta-analysis, focused on BV, and the intermediate state of dysbiosis was considered as normal in the analyses.

Nevertheless, intermediate dysbiosis has a prevalence of approximately 7%^13^, and a lack of vaginal lactobacilli may increase the risk of vaginal infection. The commonly used Nugent score of 4 implies only the half of detected intermediate vaginal microbiota that encompasses women with a reduced presence of *Lactobacillus* spp. in the vagina, and those with a complete absence of *Lactobacillus* spp. in the vagina^9^.

The limitation of this study represents a smal avaliable size of this specific group of pregnant women, e.g. Absence of vaginal lactobacilli species. Overall, given that only 1% of women with intermediate vaginal microbiota have a complete absence of detectable *Lactobacillus* spp., this small precentage makes undertaking a larger sample-sized study difficult.

In our study, we observed a decrease in the Nugent score among many participants. The greatest reduction was 4 points in subgroup C, the women who harbored no *Lactobacillus* spp. in their vagina at baseline vaginal sampling. Although the Nugent score of 0 to 3 at the follow-up visit after probiotic treatment was a favorable result, the small sample size of three resulted in this finding not being statistically significant. Subgroup B, comprising women who did not receive treatment, experienced a spontaneous decrease of the Nugent score, indicating that spontaneous regression of asymptomatic microbial disturbances can occur during pregnancy. Klebanoff et al.^18^ reported a spontaneous regression rate of 12% within 4 weeks among women with BV. In our study, among women with a Nugent score of 4, 58% of of those with and 42% of those without lactobacilli on vaginal smear experienced a spontaneous regression to a normal vaginal microbiota.

Pregnant women with a normal vaginal microbiota during the first trimester of pregnancy have a 75% lower risk of delivery before 35 weeks of gestation, and an abnormal vaginal microbiota in early pregnancy is a risk factor for PTD and low birthweight^2,4,10,12,19,20^. Moreover, a study of the vaginal microbiota of women who experienced preterm premature rupture of membranes found an increased prevalence of microbiota profiles characterized by an intermediate microbiota or a reduced dominance of *Lactobacillus* spp. with high bacterial diversity^21^.

To date, the effectiveness of antibiotic treatment of vaginal dysbiosis in pregnancy, as well as its influence on pregnancy outcomes, remains unclear^22^. The use of vaginally applied lactobacilli preparations as a simple measure for reducing the risk for PTD remains to be elucidated. We previously conducted a retrospective study and reported that the absence of vaginal lactobacilli in the first trimester of pregnancy could increase the risk of PTD in women with intermediate microbiota (OR: 0.34, 95% CI 0.21–0.55; *p* < 0.001)^13^. In this study, we conducted a prospective randomized controlled trial as a step further toward answering this question. Although we were not able to detect statistically significant differences in PTD rates among our subgroups with and without treatment, we were able to demonstrate that all three women without vaginal lactobacilli during the first trimester who were treated with Lcr regererans vaginal capsules (subgroup C) delivered at term. In contrast, the PTD rate in the seven women without vaginal lactobacilli who did not receive treatment (subgroup D) was 28.6%. Although this finding should be interpreted with caution due to the small sample size, this trend is in line with the findings of our previous study^13^.

## Conclusion

The dominance of vaginal lactobacilli is crucial to the health of every woman’s vaginal ecosystem. Their protective role is one of the fundamental characteristics of the normal microbiota, particularly during pregnancy. There is a lack of consensus on the management of vaginal dysbiosis during pregnancy. The results of this study suggest that in addition to BV and infection, it is important to address the simple absence of vaginal lactobacilli during pregnancy. The treatment options are still to be elucidated, but innovative strategies are needed. Our small study found a trend toward a benefit of treatment with vaginal lactobacilli during pregnancy, however, to evaluate this dysbiotic state and confirm these findings, further, large multicenter studies are needed to obtain an adequate sample size.

## Methods

### Setting and procedure

This prospective, randomized, observer-blinded single-center study was performed at the Medical University of Vienna, Department of Obstetrics and Gynecology, after approval by the ethics committee of the Medical University of Vienna (protocol nr.: 2258/2016) in accordance with the Declaration of Helsinki and Good Clinical Practice guidelines. The study was registered with ClinicalTrials.gov (ID: NCT02979288) on 01/12/2016.

We enrolled all pregnant women between 10 weeks plus 0 days (10 + 0) and 16 weeks plus 0 days (16 + 0) gestation who registered for a planned delivery in our department after obtaining patients’ informed consent. These women routinely underwent a prenatal consultation and screening for asymptomatic vaginal infections using the Nugent score^9^. Nugent scores of 0 to 3 were considered as normal microbiota; scores of 4 to 6 indicated an intermediate microbiota, and scores of 7–10 indicated BV. All women with a Nugent score of 4 or above underwent a follow-up smear between 20 weeks plus 0 days (20 + 0) and 22 weeks plus 0 days (22 + 0) gestation in our department. Vaginal smears were collected from the lateral vaginal wall and posterior vaginal fornix using a sterile swab. After Gram-staining the smears, they were analyzed microscopically by one of five biomedical laboratory assistants, specialized in gynecological cytopathology, at a laboratory that was certified according to the DIN EN ISO 9001:2008 quality management system standard.

### Study groups

According to the Nugent scoring system, we differentiated between two patient groups: (1) IMLN4: pregnant women with an intermediate vaginal microbiota and a Nugent score of 4 *with* lactobacilli; and (2) IM0N4: pregnant women with an intermediate vaginal microbiota and a Nugent score of 4 *without* lactobacilli. Using a computer-generated randomization code, women in both groups were assigned to either the study group (A and C) or the control group (B and D). Treatment was assigned based on the study protocol as follows: women of the study groups (subgroup A with a Nugent score of 4 *with* lactobacilli; subgroup C with a Nugent score of 4 *without* lactobacilli) were treated with one treatment cycle of mucoadhesive slow-release vaginal tablets containing 876.9 mg *Lactobacillus casei rhamnosus* (Lcr regenerans) at a physiological concentration of > 10^7^ CFU/mL. One treatment cycle included local application of two vaginal tablets on days 1 and 5. Each vaginal tablet induced a sustained release of lactobacilli for 4 days; therefore, the total treatment duration was 8 days. The study medication (Gynophilus® Protect) was authorized based on evidence of a very low individual risk of side effects and was supplied by Biose Pharmaceuticals (Arpajon-sur-Cère, France). Women in the control group (subgroup B with a Nugent score of 4 *with* lactobacilli; and subgroup D with a Nugent score of 4 *without* lactobacilli) did not receive any treatment. A vaginal follow-up smear was obtained during a routine follow-up visit, 4–6 weeks after the baseline screening smear.

### Study endpoints

The primary endpoint was restoration of the normal vaginal microbiota (Nugent score 0–3). The secondary endpoints were the incidence of PTD (defined as spontaneous birth at or before 36 + 6 gestational weeks) due to preterm premature rupture of the membranes and/or preterm labor, gestational age at delivery (recorded as term birth if delivered at or after 37 + 0 gestational weeks), neonatal birthweight, live birth rate (defined as birth of an infant with an Apgar score at 1 min of > 0), and the delivery mode (i.e., spontaneous vaginal birth, cesarean section, or instrumental vaginal delivery). As we exclusively enrolled asymptomatic pregnant women, the exclusion criteria for this study were clinical signs of a vaginal or urinary tract infection, bleeding, diarrhea, constipation, and antibiotic therapy within 4 weeks before study enrollment. Patient records and data were separated and investigator-blinded, password-protected, and de-identified prior to the analysis.

### Statistical analysis

Demographic information was summarized and displayed using descriptive statistics. The results were presented as the mean ± standard deviation, median (interquartile range), or count (%), depending on the data type and distribution. The independent samples t-test, Mann–Whitney U test, Pearson’s Chi-squared test, and Fisher’s exact test were used to assess the significance of differences between groups. Since enrollment failed to reach the expected sample size in both strata, we considered our study as exploratory and its results as hypothesis-generating. *p*-values of less than 0.05 were considered statistically significant. Statistical analysis was performed using R-Project for Statistical Computing, version 4.1.0 (R Core Team (2021). R: A language and environment for statistical computing. R Foundation for Statistical Computing, Vienna, Austria).

## Data Availability

Materials described in the manuscript, including all relevant raw data, will be freely available to any researcher wishing to use them for non-commercial purposes, without breaching participant confidentiality. Please contact coresponding author.
